# SWeeP: representing large biological sequences datasets in compact vectors

**DOI:** 10.1038/s41598-019-55627-4

**Published:** 2020-01-09

**Authors:** Camilla Reginatto De Pierri, Ricardo Voyceik, Letícia Graziela Costa Santos de Mattos, Mariane Gonçalves Kulik, Josué Oliveira Camargo, Aryel Marlus Repula de Oliveira, Bruno Thiago de Lima Nichio, Jeroniza Nunes Marchaukoski, Antonio Camilo da Silva Filho, Dieval Guizelini, J. Miguel Ortega, Fabio O. Pedrosa, Roberto Tadeu Raittz

**Affiliations:** 1 Federal University of Paraná - SEPT, Graduate Program in Bioinformatics, Curitiba, Paraná Brazil; 2 Federal University of Paraná, Department of Biochemistry and Molecular Biology, Curitiba, Paraná Brazil; 3 Federal University of Minas Gerais, Institute of Biological Sciences (ICB), Belo Horizonte, Minas Gerais Brazil; 4 Federal University of Paraná, Department of Genetics, Curitiba, Paraná Brazil; 5 Federal University of Paraná, Department of Pharmaceutical Sciences, Curitiba, Paraná Brazil

**Keywords:** Computational models, Data mining

## Abstract

Vectoral and alignment-free approaches to biological sequence representation have been explored in bioinformatics to efficiently handle big data. Even so, most current methods involve sequence comparisons via alignment-based heuristics and fail when applied to the analysis of large data sets. Here, we present “Spaced Words Projection (SWeeP)”, a method for representing biological sequences using relatively small vectors while preserving intersequence comparability. SWeeP uses spaced-words by scanning the sequences and generating indices to create a higher-dimensional vector that is later projected onto a smaller randomly oriented orthonormal base. We constructed phylogenetic trees for all organisms with mitochondrial and bacterial protein data in the NCBI database. SWeeP quickly built complete and accurate trees for these organisms with low computational cost. We compared SWeeP to other alignment-free methods and Sweep was 10 to 100 times quicker than the other techniques. A tool to build SWeeP vectors is available at https://sourceforge.net/projects/spacedwordsprojection/.

## Introduction

Biological sequence analyses and comparisons are traditionally performed using alignment algorithms, with BLAST being the most commonly used tool^1^. Even with dynamic computing techniques^2,3^, aligning large datasets requires an excessive amount of time and becomes unfeasible when complete genomes need to be analyzed^4^. Furthermore, the application of alignment techniques can become problematic when sequence identity is low because the substitution matrices can significantly affect alignment results^5^.

This need has led to the development of alternatives to accelerate structured data comparisons^6–8^. Several studies have successfully used alignment-free methods for the comparative analyses of complete genomes and other large biological sequence data sets^4–13^, but the investigation of these techniques is still necessary to ascertain their effectiveness. Therefore, approaches based on mapping relative word frequencies (k-mers) in vector spaces have been the subject of several recent studies^3–18^. Vector representations of proteins facilitate data handling and allow the use of data mining to identify important characteristics hidden in biological sequences^18–21^.

Here, we propose SWeeP, a method that handles large data sets, reducing computational costs while ensuring the quality of gene product analysis. It is based on the vector representation of protein sequences as a compact model based on the projection of k-mers sets onto a randomly oriented quasi-orthonormal base, with a sufficient number of coordinates to maintain intersequence comparisons. SWeeP uses spaced words^14^ to scan sequences and create indexes, which are employed to create a high-dimensional vector (HDV). The HDV allows dimensionality reduction upon its projection onto a lower-dimensional vector and maintains most of the comparison information, as proposed by Johnson and Lindestrauss^22^. Additionally, SWeeP has the potential to decrease the biases caused by replacement arrays^5^.

**Figure 1:**
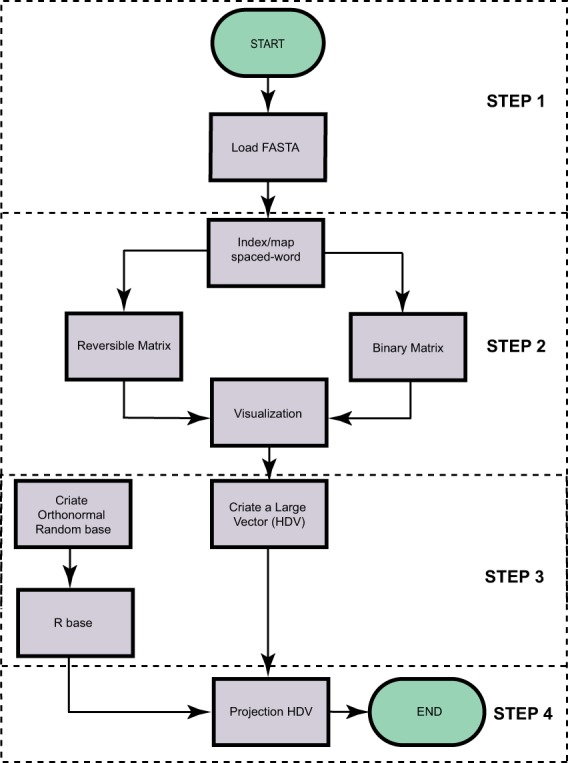
The workflow of the SWeeP processes. The process begins with uploading a multiFASTA file containing the concatenated proteins (see Supplementary Fig. S1). The squares summarize the processes of the SWeeP method. Processes are linked by arrows, illustrating their order of execution.

To demonstrate the efficiency of SWeeP, we conducted two studies, one involving whole mitochondrial protein sequences (here called mitochondrial “proteomes”) and the other involving whole bacterial proteins (bacterial “proteomes”). We constructed phylogenetic trees of the mitochondrial proteomes and compared the performance of the alignment and alignment-free methods. We also created SWeeP representations of all available complete bacterial genomes using their protein sequences. We then isolated the representations of the bacterial genera of some model organisms (*Corynebacterium*, *Klebsiella*, and *Escherichia)* and developed a machine learning approach to demonstrate the classification capacity of the general *SWeeP* model. The flowchart outlining the SWeeP model processes is shown in Fig. 1.

## Results

### SWeeP

The input for SWeeP consists of a multiFASTA file containing amino acid sequences. In the case of a proteome containing several sequences (e.g., several genes), the proteins are concatenated, separated by delimiters - which are ignored in the construction of the vectors - to form a single sequence for each protein set (See Supplementary Fig. S1). Each proteome is then represented by a two-dimensional matrix *M*, of spaced words from the entire concatenated sequence using a predefined mask. Matrix *M* can be constructed in two formats: (1) reversible, based on the geometric mean of prime numbers, and (2) binary, which is economical and rapidly processed. The binary form was used for the case studies described in this paper.

Matrix *M*, when linearized by columns, is a vector reflecting highly representative sequence data, which we designate as a higher-dimensional vector (HDV). The SWeeP model projects these HDVs onto a quasi-orthonormal base, according to the Johnson–Lindestrauss lemma^22^, aiming to enable efficient performance with large data sets.

The following steps are used to convert amino acid (*aa*) sequences to SWeeP vectors.

For a given amino acid sequence $\,({S}_{i})$, defined in 1:

1 $$({S}_{i}),\,{S}_{i}\in \,\{{\rm{A}},{\rm{R}},{\rm{N}},{\rm{D}},{\rm{C}},{\rm{Q}},{\rm{E}},{\rm{G}},{\rm{H}},{\rm{I}},{\rm{L}},{\rm{K}},{\rm{M}},{\rm{F}},{\rm{P}},{\rm{S}},{\rm{T}},{\rm{W}},{\rm{Y}},{\rm{V}}\},\,{\rm{representing}}\,{\rm{the}}\,{\rm{amino}}\,{\rm{acids}}.$$

$$i\,{\epsilon }\,I=[1,\,n]$$

Let $\bar{N}$ be an integer pointing uniquely to a subsequence of $({S}_{i})$ of length k according to a given indexing function *f*:

2 $$\bar{N}=f({({S}_{k})}_{k\le n})$$

We propose a reversible indexing function (*f*) to relate moving windows in $({S}_{i})$ to coordinates in a matrix. Function f (Eq. 3) uniquely addresses the sequence in a given window, aiming to map all possible spaced k-mers to a matrix. Various functions could be used for this purpose; we chose the enumeration of the amino acid sequences starting from 1.

3 $$f(({S}_{k}))=1+\mathop{\sum }\limits_{q=1}^{k}\,V({S}_{q}-1)\,\ast \,{(20)}^{(q-1)}$$

As *f* is reversible, for any finite $\,\bar{N}$, there is a unique corresponding valid sequence, ${({S}_{r})}_{r\le n}$, of length r (Eq. 4):

4 $${f}^{-1}\,(\bar{N},{\rm{r}})={({S}_{r})}_{r\le n}$$

As an example for *f*, consider *S*_*K*_ representing an integer ≥1 in a vigesimal system (base 20), with *k* digits.

V is given in the correspondence list below:

$$\begin{array}{lllllllllllllllllllll}aa & {\rm{A}} & {\rm{R}} & {\rm{N}} & {\rm{D}} & {\rm{C}} & {\rm{Q}} & {\rm{E}} & {\rm{G}} & {\rm{H}} & {\rm{I}} & {\rm{L}} & {\rm{K}} & {\rm{M}} & {\rm{F}} & {\rm{P}} & {\rm{S}} & {\rm{T}} & {\rm{W}} & {\rm{Y}} & {\rm{V}}\\ V & 1 & 2 & 3 & 4 & 5 & 6 & 7 & 8 & 9 & 10 & 11 & 12 & 13 & 14 & 15 & 16 & 17 & 18 & 19 & 20\end{array}$$

It can be seen that the order in which the amino acids are enumerated has no effect on the geometric properties of the vectors, but rather only to point to their coordinates (the same vectors for any order in V). Thus, we do not assign biological meaning to the numbers that are instead used to point to “boxes” containing information regarding the sequences (k-mers), as referends in other approaches^13–23^.

E.g.: For a subsequence composed of the amino acids ML:

$$f(ML)=1+(V(M)-1)\,\ast \,{20}^{0}+(V(L)-1)\,\ast \,{20}^{1}=1+(13-1)\,\ast \,{20}^{0}+(11-1)\,\ast \,{20}^{1}=213$$

and

$${f}^{-1}(213,2)=ML$$

Now, we define Z (Eq. 5) as coordinates representing a spaced window in (S_i_):

5 $${Z}_{i}=(X,Y)=(f\,(({s}_{p})),\,f\,(({s}_{q})))$$

(S_p_) and (S_q_) are subsequences of (S_i_), and *p* and *q* are the intervals:

$$p=[i,\,(i+{\rm{\alpha }}-1)]$$

$$q=[(i+{\rm{\alpha }}+{\rm{\gamma }}),\,(i+{\rm{\alpha }}+{\rm{\gamma }}+{\rm{\beta }}-1)]$$

Here, a spaced window is a moving window controlled by a mask consisting of “*take”* (ones) and “*don’t care”* (zeros) positions^23^. The region between p and q, with zeros in the mask, is given by g; α, β and γ are the lengths of *p*, *q*, and *g* respectively (e.g., 111000011, α = *3*, β = *2*, and γ = *4*; *k* = *9*).

The content of each set of coordinates (*X*, *Y*) in the matrix is calculated in a form that enables the retrieval of all the initial positions in a sequence where the windows corresponding to the coordinates are found. To do this, we exploit the *unique factorization theorem* (or *fundamental theorem of arithmetic*), which states that any integer number is uniquely represented by the product of a set of prime numbers.

We define *P* as an ordered subset of consecutive prime numbers $\,{P}_{I}$:

$${P}_{I}=\{2,3,5,7,\ldots ,{P}_{n}\}$$

For instance,

$${P}_{(i=1)}=2,\,{P}_{(i=3)}=5,\ldots $$

Let us consider the set of positions in (S_i_), where $\,{Z}_{i{\epsilon }J}$, is related to the same coordinates ${Z}_{j}$= $({X}^{\text{'}},{Y}^{\text{'}})$. All *i* positions related to ${Z}_{j}$ can be reversibly represented by the geometric mean of the prime numbers $\,{P}_{j}$, to the power of a rational constant $\varepsilon$:

6 $$G({Z}_{j})=G(X^{\prime} ,Y^{\prime} )=\prod _{J}\,{P}^{\varepsilon /{\rm{\lambda }}}$$

where λ, is the number of elements in *J*, and 0 < *ε* ≤ 1.

Now, we can define *M* as *Mr* representing (*S*_*i*_) in a reversible manner:

7 $$Mr\,({\rm{X}},{\rm{Y}})=\{\begin{array}{ll}G, & (X,Y)\in {Z}_{J}\\ 0, & \,otherwise\end{array}$$

Or as a binary compact matrix representing $\,({S}_{i})$ with $\varepsilon =0$:

8 $$Mb\,({\rm{X}},{\rm{Y}})=\{\begin{array}{ll}1, & (X,Y)\in {Z}_{i}\\ 0, & otherwise\end{array}$$

When $\varepsilon \to 0$, $Mr=Mb$. This means that, mathematically, there is a reversible vector (HDV) associated with $Mr$ that is very close to a binary vector associated with $Mb$. The larger $\varepsilon$, the higher the impact of the sequence k-mers relative positions on the vector representation.

#### Projection

An orthonormal base is a set of orthogonal vectors. The projection of a set of vectors onto an orthonormal base creates a representation of the set in the given base. This projection is the product of the matrix of vectors to be projected and the matrix of the base. A *quasi-orthonormal* base refers to a base that is sufficiently orthogonal to obtain a satisfactory projection at a reasonable computational cost. In this case, the sufficient orthogonality condition is that the internal product of the vectors of the base is sufficiently small, but not necessarily zero. In this study, we used *quasi-orthonormal* bases to obtain the projections for SWeeP.

We constructed a random bases, *R*_*s*_ (SRB: SWeeP Random Base) to obtain the SWeeP projections of *W*, the matrix product:

9 $${W}_{s}=W{R}_{s}$$

In Eq. 9, the subscript *s* denotes the number of coordinates defined in base *R*_*s*_.

Here, we obtained R through economy-size Single Value Decomposition (SVD) of a random vector *B* of lengths *u* × *v* where *u* is the number of coordinates in the original space and *v* the number of coordinates in the projection. Note that we propose *v* ≪ *u* (*u* = 160,000 and *v* = 600 in the cases studied in this paper), and consequently that SVD of *B* be computationally simpler than set of vectors of length *u* e.g. *W*.

Vector *W*_*s*_ has a smaller dimensionality than W, but with a sufficiently large *s*, the projection conserves the spatial comparability of the instances represented in W. Naturally, the SRB must be kept for future tasks involving the inclusion of new sequences in the analyses - a common event in data mining. The SRBs employed in this article were developed in MATLAB® and are available for download (see Data Availability). An example of these processes is available in Supplementary Fig. S2.

### Study 1: Representation of mitochondrial proteomes

The included studies all use protein data; nonetheless, nucleotide sequences can be used in an analogous manner. All mitochondrial protein sequences available in RefSeq^24^, from all complete mitochondrial genomes were represented in SWeeP vectors.

**Figure 2:**
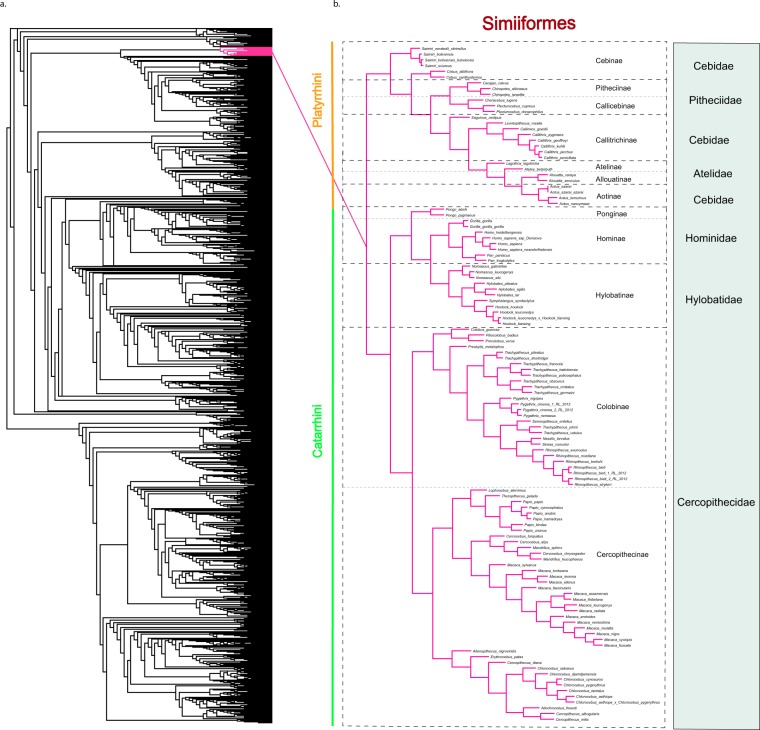
Representation of Primates in the global mitochondrial tree created using SWeeP. (**a)** A cladogram of the SWeeP global mitochondrial tree containing 8,426 proteomes, with the projection size of 600 coordinates for the neighbor joining model. Pink: the position of primates in the global tree. (**b)** An enlarged branch phylogram, containing Primates. In blue square, the families; In dotted square, the subfamilies; In orange, the Platyrrhini parvorder; In green, the Catarrhini parvorder. The rest of the primates are shown in Supplementary Fig. S3.

After downloading the sequences, we concatenated the proteins, where each proteome was represented by a SWeeP vector with the projection defined in the configuration test (see Implementation – Definition of parameters). Phylogenetic trees of the entire set of 8426 mitochondrial proteomes were constructed using the neighbor-joining and unweighted pair group method with arithmetic mean (UPGMA). The complete trees are available (see Data Availability). We chose to focus on Primate families because their mitochondrial proteins are manually curated and available in the literature^25^, and these organisms’ evolutionary history is well-defined (Fig. 2).

We analyzed several branches of the trees, but herein, we will only show the primate branches. The Platyrrhini and Catarrhini parvorders were separated as expected. The inner divisions in the Catarrhini branch are also in accord with the literature, what is clearly shown in the Hominid branches whose organization is in agreement with other studies^26–28^.

We found that, unlike reports in the literature^29,30^, the Cebidae family is not monophyletic in the SWeeP tree. A similar result was observed when the Platyrrhini branch was analyzed with two other approaches (Clustal Omega^31^ and Prot-Spam^13^), which suggests the need for further studies on the mitochondrial proteomes data of this branch.

We also present the branches containing the other Primates in the global tree, in Supplementary Fig. S3.

#### Performance test

We defined the vector construction time for the 100- to 3000-coordinate projections and without a size reduction (160 K) as the time spent from the moment the multiFASTA file was read until termination. We observed that this time varied between a few seconds and minutes, as seen in Supplementary Table S1. The processing time was measured for each projection size at increments of 200 coordinates, including the unreduced projection *W* (160 K coordinates). Projection processing is rapid, and the processing time grows linearly as the projection increases, ranging from 8 seconds to 4.36 minutes. Estimation of the time for the construction of the mitochondrial phylogenetic trees from the projections is also rapid: approximately 10 seconds.

#### Comparison between SWeeP and alignment methods

**Figure 3:**
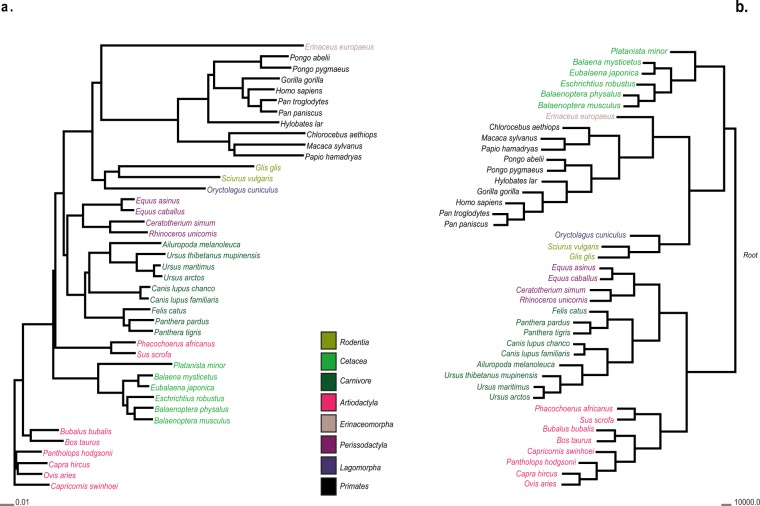
Comparison between phylogenetic trees constructed using Clustal Ômega and SWeeP. Neighbor-joining trees constructed using the mammalian proteome dataset^8^. All proteins were concatenated in the following order: NADH1, NADH2, COX1, COX2, ATP8, ATP6, COX3, NADH3, NADH4L, NADH4, NADH5, NADH6, and CYTB. The mammalian orders are represented by the colors in the trees: Rodentia, Cetacea, and Carnivore in shades of green; Artiodactyla in pink; Erinaceomorpha, Perissodactyla, and Lagomorpha in shades of purple; Primates in black. (**a)** Tree built with Clustal Omega using standard parameters (online version). (**b)** The tree constructed using a 600 coordiante SWeeP projection.

We compared phylogenetic trees of a mitochondrial proteome dataset from 41 mammals^8^ constructed with SWeeP and Clustal Omega software^31^ (Fig. 3). We chose a smaller data subset because it is not possible to perform multiple alignment of the entire set of mitochondrial data in Clustal Omega (8,426 proteomes). To carry out the comparison, we organized all the mammalian mitochondrial proteins in an identical order; however, this is not necessary for SWeeP.

The Clustal Omega method took 93 seconds, whereas SWeeP took 2 seconds, indicating that our method is not only quicker but also more practical since there is no need to align or even order the protein sequences to process them. The phylogenetic trees produced by the SWeeP vectors show a better organization than those produced by the alignment-based technique. For example, in the tree built by the Clustal Omega, there is a division in the branch containing the Artiodactyla family while in the tree constructed by SWeeP, the distribution of families is taxonomically correct.

As mentioned above, it was not possible to align the entire mitochondrial dataset with Clustal Omega. This makes SWeeP a better option for comparing large datasets due its better effectiveness, accuracy and lower computational cost than alignment techniques.

#### Comparison of alignment free methods

We compared SWeeP to other alignment-free methods by using the mitochondrial proteome sequences. For these tests, we used the standard parameters for our approach, i.e., k = 5 (4 *take* and 1 *don’t care* positions - “11011” mask). To select the tools for comparison, we use the data available in Supplementary Table S2 and the following inclusion criteria: a. Publicly available; b. Not only useful for phylogeny; c. Accepts input files in amino acid format; d. Published in the last 5 years (for criteria details, see Supplementary Table S3).

The performance tests were conducted on an ordinary computer, to evaluate the performance of tools with lower processing power (for the specifications, see Implementation). Prot-Spam^13^, BioVec^21^ and Kmacs^32^ were tested. It is important to note that only the SWeeP algorithm is based on a vectoral representation of biological sequences and dimensionality reduction these tools cannot be considered similar to SWeeP. We tested each tool using its default parameters, Table 1 lists whether or not the tools use a vector representation as well as the time required to construct the distance matrix and output.

**Table 1:** Comparison among alignment free methods.

Tools	Vector representation (Y/N)	Distance matrix construction time (min)	Output
SWeeP	Y	6,4	Vector
Prot-SpaM	N	60,6	Distance Matrix
Kmacs	N	564,3	Distance Matrix
BioVec	Y*	60,4	Distance Matrix Neural network Database

Note: Y- Yes and N- No. *The vector was obtained by writing custom scripts.

None of the tested tools provided vectors as output. The Biovec output is a Distance Matrix, a neural network model, and a database containing the reported data. We were able to write a Python script to obtain the output as vectors, though this is not provided by the application. The final outputs of Kmacs and Prot-Spam is a distance matrix of mismatches (for Kmacs) and matches (for Prot-Spam). Prot-Spam output is saved as a “.DMat” (default) file. To use the matrix of Kmacs, it is necessary to convert the data to the Phylip format, which limits the utility of the application.

Representation of the nucleotide and/or amino acid sequences in vector form is essential for mathematical, statistical, and computational analysis. Here, the vectoral representation allows transformation of the symbolic representation into a structured numerical representation, which allows its use in machine learning algorithms and for knowledge discovery in biological data. The construction of the distance matrix is fundamental for wide context comparisons, particularly in scenarios involving thousands of sequences. In our tests SWeeP was 10 times faster than ProtSpam and BioVec and 100 times faster than Kmacs.

SWeeP is applicable to general purpose data mining and sequence comparison. Construction of phylogenetic trees was used as an example to explore the comparability potential of the method. We manually validated the taxonomical consistency of the phylogenetic trees based on the available literature.

SWeeP was quicker than BioVec, Prot-Spam, and Kmacs while constructing high quality trees that we considered equivalent; however, a detailed comparison of the trees is not within the scope of this paper. The trees constructed by Prot-Spam, BioVec, and Kmacs are available upon request.

### Study 2: Representation of bacterial proteomes

A SWeeP vector with a 600-coordinate projection was created for the coding sequences (CDS) of all complete bacterial genomes available from NCBI at the time of writing (10,324 microorganisms) and a phylogenetic tree was constructed from them using the Ward method^33^. In this case study, the SWeeP method proved to be computationally effective when applied to a large number of proteomes of considerable size. The global phylogenetic tree of the bacterial taxa was analyzed manually, and the results were consistent with trees reported in the literature^34–36^. To the best of our knowledge, there is no other analogous comparison among these organisms that has been automatically created solely from complete genome sequence data.

Another goal of this analysis was to obtain a graphical visualization and detailed comparison of the genomes for *Corynebacterium*, *Klebsiella* and *Escherichia*. We also chose specific strains of these model organisms, *Escherichia coli* strains K12 and CFT073, *Klebsiella pneumoniae* HS11286, *Klebsiella variicola* AT-22, *Corynebacterium pseudotuberculosis* C231, and *Corynebacterium ulcerans* 809, (Supplementary Table S4) for analysis.

**Figure 4:**
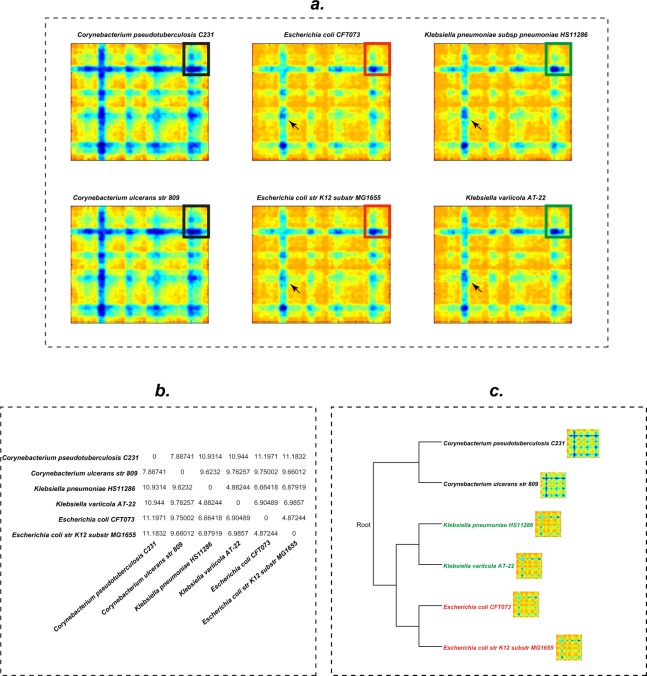
Graphic representation of the bacterial proteome. (**a)** Heatmaps of bacterial proteomes represented by Matrix M (400 × 400). Black squares highlighted in the bacterial proteome depictions show one of the similarity areas between the proteomes of the *Corynebacterium* strains. Red squares show one of the similarities between the proteomes of *Escherichia coli* strains. Green squares show similarity areas between *Klebsiella* species. The arrows point to regions with less similarity between the *Escherichia coli* and *Klebsiella* proteomes. (**b**) Euclidean distance between the 600-coordinate projections of the bacterial proteomes in a. (**c**) The phylogenetic tree of bacteria created by a 600-coordinate SWeeP projection.

A binary *M*_*b*_ matrix and the respective 600-coordinate SWeeP projection were created for each microorganism. Figure 4a depicts heatmaps of the matrices; each column corresponds to microorganisms of the same genus (one genus per column). One can see that microorganisms of the same genus are more similar to each other than to organisms in different genera. Nonetheless, zooming in for more detail (rectangle) reveals that even microorganisms belonging to the same genus differ from each other. This effect becomes clearer when the distance matrix (Fig. 4b) and the dendrogram (Fig. 4c) constructed from the SWeeP distance matrix of the six microbes are compared. Phylogenetically closer microorganisms show smaller reciprocal distances and greater proximity in the dendrogram.

From the 10324 bacterial proteomes projected with SWeeP onto 600 coordinates, we selected 1001 organisms and classified them as 1 – *Corynebacterium, 2 - Klebsiella* and 3 – *Escherichia*. A training set with 700 instances was used to construct a Support Vector Machine (SVM) trained in Weka software^37^. We then tested the trained model in the remaining 301 instances. The results are shown in Supplementary Fig. S4. In this test, all the instances of three genera were correctly classified. It can be seen that although the differences between *Escherichia* and *Klebsiella* are difficult to notice (Fig. 4a), these were classified properly through machine learning.

**Figure 5:**
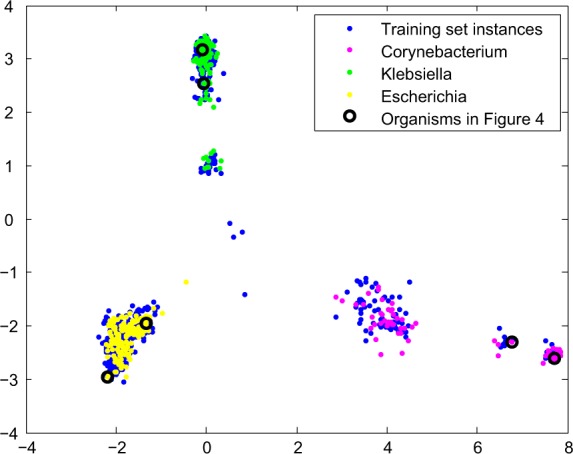
Analysis of two principal components of the SWeeP projection for the three genera of model organisms. The figure highlights the comparability potential of the information contained in the SWeeP projection with 600 coordinates. In blue the points of the training set (used to create the base of the PCA). In pink, yellow and green the test set points. The black circles mark the comparative instances of Fig. 4.

Additionally, for data visualization, the two principal components of the principal component analysis (PCA) of the vectors from the training set were plotted for each instance in the training and test sets (Fig. 5). Here, it can be seen that all the three genera are separated consistently, including the organisms referenced in Fig. 4 (black circles in Fig. 5).

It would be computationally difficult to perform PCA with the complete SWeeP matrix (160k) because of the high dimensionality, which in the case of the “11011” mask would require at least 2 × 10^11^ bytes of memory, that is, approximately 190 GB. In contrast, SWeeP projection onto 600-coordinates only requires 0.027 GB. Thus, we propose a viable approach to PCA for large volumes of sequences.

## Discussion

Although alignment techniques are suitable for the comparison of protein sequences, their application to the analysis of large volumes of data is limited. In this study, we applied the SWeeP method to analyze large numbers of proteome sequences. We compared SWeeP to other methods, and for the first time, automatically constructed phylogenetic trees from complete mitochondrial and bacterial proteomes. We also used SWeeP for machine learning and PCA, showing its effectiveness in these applications.

The SWeeP method enables the rapid and sensitive construction of compact phylogenetic trees. The mitochondrial proteome trees created using SWeeP are currently the most complete trees available, including the supertrees for mitochondrial data. We show that alternative approaches such as SWeeP can be superior to sequence alignment–based techniques. We propose that the SWeeP method is an efficient alternative to sequence comparisons of large datasets. It should be noted that although other alternative sequence comparison techniques exist, sequence alignment is still the preferred method for most biological sequence comparisons. SWeeP is a new, innovative alignment-free method that could supersede the traditional alignment techniques in the comparison of biological sequences.

Vector representation of information is universally used and is well-defined in most areas. We propose this new approach so that available mathematical and computational resources can be extended and applied more easily to the analysis and mining of biological sequences.

## Implementation

The SWeeP specific functions used in this study were implemented using the MATLAB programming language. The steps presented in the definition of parameters for study 1 and study 2 were performed on a 40-core Intel Xeon processor with 256 GB of RAM running Ubuntu 16.04.1 LTS. The comparisons with alignment-free methods were performed on an Intel Core i5 processor with 16 GB RAM running Biolinux 8.0 (based on Ubuntu 18.04.01 LTS). The implementation is freely available for both operating systems (see Data Availability). For machine learning tests, we used Weka Software^37^.

### Test set

The mitochondrial protein sequences were obtained from the RefSeq database available at ftp://ftp.ncbi.nlm.nih.gov/refseq/release/. Visualization and manipulation of the phylogenetic trees constructed using SWeeP were performed using Dendroscope 3^38^.

The CDSs of the bacterial genomes used for the graphical representation of the matrix *M* were obtained from NCBI. The organisms and their respective accession numbers are given in Supplementary Table S4.

### Definition of parameters

In this study, a reversible matrix is one where 1 is taken for ***ε*** ($\varepsilon =1$) and a binary matrix is one where 0 is taken for $(\varepsilon \to 0$). For all examples and case studies, *W* (higher-dimensional vectors) were obtained from a binary matrix. This choice is due to the fact that the most relevant metric in this study is computational feasibility (SWeeP’s compression and processing speed) combined with our test findings showing that SWeeP (600 coordinate projection) is highly correlated with W with a rate of 0.98 and p-value < 0.01. The *spaced words* were selected by applying the “11011” mask in all cases because it is suggested by the literature to be a good choice for proteins^23^. Nevertheless, the SWeeP method is adjustable and allows for several projections and multiple k-mers (via the alteration and/or addition of masks), enabling the model to be fitted to the data to be mined^14,15^, as increased k-mer size increases computational difficulty.

The best distance metric for mitochondrial proteomes is Euclidean distance, and the 600-coordinate sized projection was chosen for the analysis after manual validation and plot analysis (see Supplementary Fig. S5 and Table S5).

## Supplementary information

Supplementary information

## Data Availability

https://sourceforge.net/projects/spacedwordsprojection/.
