# Supplementary material for: SWeeP: representing large biological sequences datasets in compact vectors
Source: Sci Rep. 2020 Jan 9;10:91. doi: 10.1038/s41598-019-55627-4 (PMC6952362; doi:10.1038/s41598-019-55627-4)
Supplement: Supplementary file 1 — Supplementary information [file 41598_2019_55627_MOESM1_ESM.pdf]

SUPPLEMENTARY MATERIAL

SWeeP: Representing large biological sequences datasets in compact vectors

Camilla Reginatto De Pierri[1,2†], Ricardo Voyceik[3†], Letícia Graziela Costa Santos de Mattos[1], Mariane Gonçalves Kulik[1], Josué Oliveira Camargo[1,2], Aryel Marlus Repula de Oliveira[1,4], Bruno Thiago de Lima Nichio[1,2], Jeroniza Nunes Marchaukoski[1], Antonio Camilo da Silva Filho[1,5], Dieval Guizelini[1], José Miguel Ortega[3], Fabio de Oliveira Pedrosa[1,2], Roberto Tadeu Raittz[1,3,4]*

[1]Federal University of Paraná, SEPT, Graduate Program in Bioinformatics, Curitiba, Paraná, Brazil.

[2]Federal University of Paraná, Department of Biochemistry and Molecular Biology, Curitiba, Paraná, Brazil.

[3]Federal University of Minas Gerais, Institute of Biological Sciences (ICB), Belo Horizonte, Minas Gerais, Brazil.

[4]Federal University of Paraná, Department of Genetics, Curitiba, Paraná, Brazil.

[5]Federal University of Paraná, Department of Pharmaceutical Sciences, Curitiba, Paraná, Brazil.

*raittz@gmail.com

[†]These authors contributed equally to this work

```
>Homo_sapiens
MPMANLLLLIVPILIAMAFLMLTERKILGYMQLRKGPNVVGPYGLLQPFADAMKLFTKEPLKPATSTITL
YITAPTLALTIALLLWTPLPMPNPLVNLNLGLLFILATSSLAVYSILWSGWASNSNYALIGALRAVAQTI
SYEVTLAIILLSTLLMSGSFNLSTLITTQEHLWLLLPSWPLAMMWFISTLAETNRTPFDLAEGESELVSG
FNIEYAAGPFALFFMAEYTNIIMMNTLTTTIFLGTTYDALSPELYTTYFVTKTLLLTSLFLWIRTAYPRF
RYDQLMHLLWKNFLPLTLALLMWYVSMPITISSIPPQT*****MNPLAQPVIYSTIFAGTLITALSSHWF
FTWVGLEMNMLAFIPVLTKKMNPRSTEAAIKYFLTQATASMILLMAILFNNMLSGQWTMTNTTNQYSSLM
IMMAMAMKLGMAPFHFWVPEVTQGTPLTSGLLLLTWQKLAPISIMYQISPSLNVSLLLTLSILSIMAGSW
GGLNQTQLRKILAYSSITHMGWMMAVLPYNPNMTILNLTIYIILTTTAFLLLNLNSSTTTLLLSRTWNKL
TWLTPLIPSTLLSLGGLPPLTGFLPKWAIIEEFTKNNSLIIPTIMATITLLNLYFYLRLIYSTSITLLPM
SNNVKMKWQFEHTKPTPFLPTLIALTTLLLPISPFMLMIL*****MFADRWLFSTNHKDIGTLYLLFGAW
AGVLGTALSLLIRAELGQPGNLLGNDHIYNVIVTAHAFVMIFFMVMPIMIGGFGNWLVPLMIGAPDMAFP
RMNNMSFWLLPPSLLLLLASAMVEAGAGTGWTVYPPLAGNYSHPGASVDLTIFSLHLAGVSSILGAINFI
TTIINMKPPAMTQYQTPLFVWSVLITAVLLLLSLPVLAAGITMLLTDRNLNTTFFDPAGGGDPILYQHLF
WFFGHPEVYILILPGFGMISHIVTYYSGKKEPFGYMGMVWAMMSIGFLGFIVWAHHMFTVGMDVDTRAYF
TSATMIIAIPTGVKVFSWLATLHGSNMKWSAAVLWALGFIFLFTVGGLTGIVLANSSLDIVLHDTYYVVA
HFHYVLSMGAVFAIMGGFIHWFPLFSGYTLDQTYAKIHFTIMFIGVNLTFFPQHFLGLSGMPRRYSDYPD
AYTTWNILSSVGSFISLTAVMLMIFMIWEAFASKRKVLMVEEPSMNLEWLYGCPPPYHTFEEPVYMKS*****
MAHAAQVGLQDATSPIMEELITFHDHALMIIFLICFLVLYALFLTLTTKLTNTNISDAQEMETVWTI
LPAIILVLIALPSLRILYMTDEVNDPSLTIKSIGHQWYWTYEYTDYGGLIFNSYMLPPLFLEPGDLRLLD
VDNRVVLPIEAPIRMMITSQDVLHSWAVPTLGLKTDAIPGRLNQTTFTATRPGVYYGQCSEICGANHSFM
PIVLELIPLKIFEMGPVFTL*****MPQLNTTVWPTMITPMLLTLFLITQLKMLNTNYHLPPSPKPMKMK
NYNKPWEPKWTKICSLHSLPPQS*****MNENLFASFIAPTILGLPAAVLIILFPPLLIPTSKYLINNRL
ITTQQWLIKLTSKQMMTMHNTKGRTWSLMLVSLIIFIATTNLLGLLPHSFTPTTQLSMNLAMAIPLWAGT
VIMGFRSKIKNALAHFLPQGTPTPLIPMLVIIETISLLIQPMALAVRLTANITAGHLLMHLIGSATLAMS
TINLPSTLIIFTILILLTILEIAVALIQAYVFTLLVSLYLHDNT*****MTHQSHAYHMVKPSPWPLTGA
LSALLMTSGLAMWFHFHSMTLLMLGLLTNTLTMYQWWRDVTRESTYQGHHTPPVQKGLRYGMILFITSEV
FFFAGFFWAFYHSSLAPTPQLGGHWPPTGITPLNPLEVPLLNTSVLLASGVSITWAHHSLMENNRNQMIQ
ALLITILLGLYFTLLQASEYFESPFTISDGIYGSTFFVATGFHGLHVIIGSTFLTICFIRQLMFHFTSKH
HFGFEAAAWYWHFVDVVWLFLYVSIYWWGS*****MNFALILMINTLLALLLMIITFWLPQLNGYMEKST
PYECGFDPMSPARVPFSMKFFLVAITFLLFDLEIALLLPLPWALQTTNLPLMVMSSLLLIIILALSLAYE
WLQKGLDWTE*****MPLIYMNIMLAFTISLLGMLVYRSHLMSSLLCLEGMMLSLFIMATLMTLNTHSLL
ANIVPIAMLVFAACEAAVGLALLVSISNTYGLDYVHNLNLLQC*****MLKLIVPTIMLLPLTWLSKKHM
IWINTTTHSLIISIIPLLFFNQINNNLFSCSPTFSSDPLTTPLLMLTTWLLPLTIMASQRHLSSEPLSRK
KLYLSMLISLQISLIMTFTATELIMFYIFFETTLIPTLAIITRWGNQPERLNAGTYFLFYTLVGSLPLLI
ALIYTHNTLGSLNILLLTLTAQELSNSWANNLMWLAYTMAFMVKMPLYGLHLWLPKAHVEAPIAGSMVLA
AVLLKLGGYGMMRLTLILNPLTKHMAYPFLVLSLWGMIMTSSICLRQTDLKSLIAYSSISHMALVVTAIL
IQTPWSFTGAVILMIAHGLTSSLLFCLANSNYERTHSRIMILSQGLQTLLPLMAFWWLLASLANLALPPT
INLLGELSVLVTTFSWSNITLLLTGLNMLVTALYSLYMFTTTQWGSLTHHINNMKPSFTRENTLMFMHLS
PILLLSLNPDIITGFSS*****MTMHTTMTTLTLTSLIPPILTTLVNPNKKNSYPHYVKSIVASTFIISL
FPTTMFMCLDQEVIISNWHWATTQTTQLSLSFKLDYFSMMFIPVALFVTWSIMEFSLWYMNSDPNINQFF
KYLLIFLITMLILVTANNLFQLFIGWEGVGIMSFLLISWWYARADANTAAIQAILYNRIGDIGFILALAW
FILHSNSWDPQQMALLNANPSLTPLLGLLLAAAGKSAQLGLHPWLPSAMEGPTPVSALLHSSTMVVAGIF
LLIRFHPLAENSPLIQTLTLCLGAITTLFAAVCALTQNDIKKIVAFSTSSQLGLMMVTIGINQPHLAFLH
ICTHAFFKAMLFMCSGSIIHNLNNEQDIRKMGGLLKTMPLTSTSLTIGSLALAGMPFLTGFYSKDHIIET
ANMSYTNAWALSITLIATSLTSAYSTRMILLTLTGQPRFPTLTNINENNPTLLNPIKRLAAGSLFAGFLI
TNNISPASPFQTTIPLYLKLTALAVTFLGLLTALDLNYLTNKLKMKSPLCTFYFSNMLGFYPSITHRTIP
YLGLLTSQNLPLLLLDLTWLEKLLPKTISQHQISTSIITSTQKGMIKLYFLSFFFPLILTLLLIT*****
MMYALFLLSVGLVMGFVGFSSKPSPIYGGLVLIVSGVVGCVIILNFGGGYMGLMVFLIYLGGMMVVFGYT
TAMAIEEYPEAWGSGVEVLVSVLVGLAMEVGLVLWVKEYDGVVVVVNFNSVGSWMIYEGEGSGLIREDPI
GAGALYDYGRWLVVVTGWTLFVGVYIVIEIARGN*****MTPMRKTNPLMKLINHSFIDLPTPSNISAWW
NFGSLLGACLILQITTGLFLAMHYSPDASTAFSSIAHITRDVNYGWIIRYLHANGASMFFICLFLHIGRG
LYYGSFLYSETWNIGIILLLATMATAFMGYVLPWGQMSFWGATVITNLLSAIPYIGTDLVQWIWGGYSVD
SPTLTRFFTFHFILPFIIAALATLHLLFLHETGSNNPLGITSHSDKITFHPYYTIKDALGLLLFLLSLMT
LTLFSPDLLGDPDNYTLANPLNTPPHIKPEWYFLFAYTILRSVPNKLGGVLALLLSILILAMIPILHMSK
QQSMMFRPLSQSLYWLLAADLLILTWIGGQPVSYPFTIIGQVASVLYFTTILILMPTISLIENKMLKWA*****
```

Supplementary Figure S1 | Protein concatenation. Concatenation of mitochondrial proteins in the *Homo sapiens* proteome. Highlighted pink asterisks are used to link protein sequences to form a single sequence, representing a proteome. Proteins from all 8,426 organisms in the dataset were concatenated in this manner. This step is optional and can be performed if the user deems it necessary.

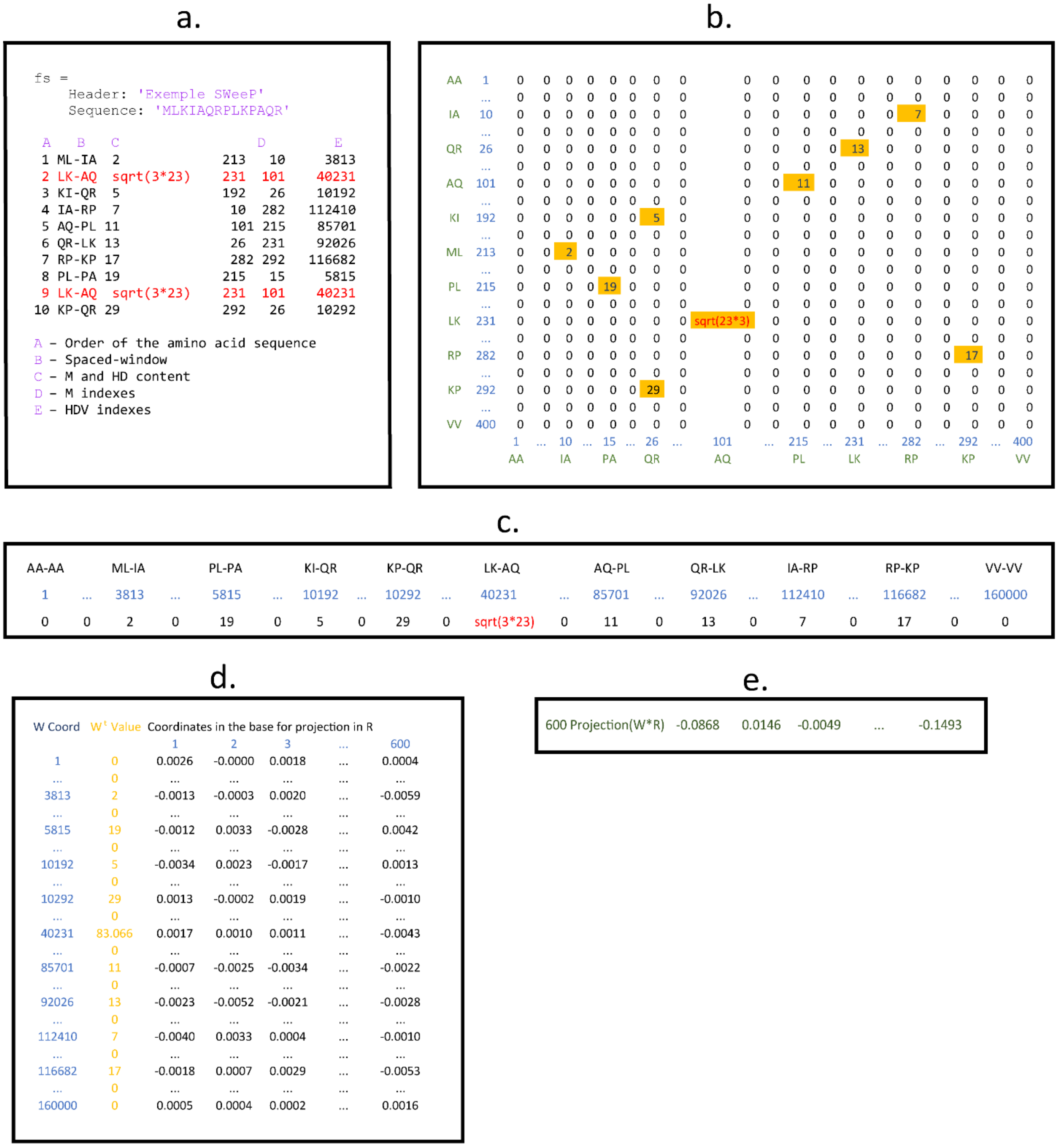

Supplementary Figure S2 – Diagram of transformation from a fasta sequence to a SWeeP projection in 600 coordinates | a. Correspondence table of spaced kmers and its contents and respective indexes in M and HDV, for the mask 11011 and ε=1; **b.** view of filled regions in M (equation 7); **c.** view of filled regions in HDV; **d.** schematic view of SWeeP projection for HDV/W in a 600 coordinates R base; **e.** representation of matrix product – SWeeP vector with 600 coordinates.

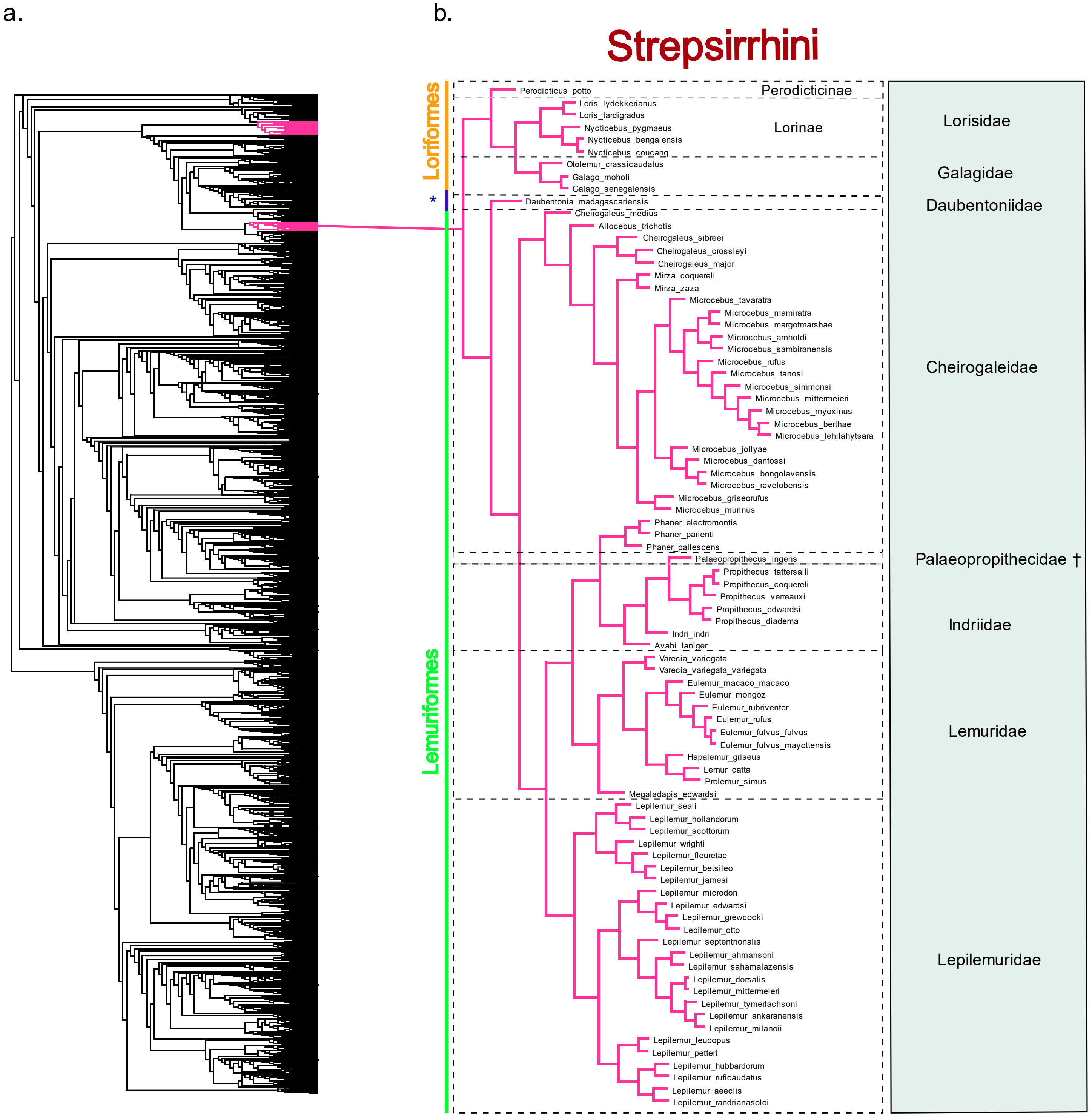

Supplementary Figure S3 | The suborder Strepsirrhini: Lemuriformes (green), Chiromyiformes (blue *) and Loriformes (orange) infraorder branches of Primates. a. The cladogram containing 8,426 mitochondrial proteomes, which was generated with a projection size of 600 for the neighbor-joining model. Primates are highlighted in pink. b. The branch containing the remaining Primates. In the blue square are represented the families; †the Palaeopropithecidae family is extinct, according to Junguers et al. (1997)[1]. Only the Lorisidae family has subfamily representation (Perodicticinae and Lorinae).

```
=== Run information ===

Scheme:      weka.classifiers.functions.LibSVM -S 0 -K 2 -D 3 -G 0.0 -R 0.0 -N 0.5 -M 40.0 -C 1.0 -E 0.001 -P 0.1 -model "C:\\Program Files\\Weka-3-8" -seed 1
Relation:    VariavelMatlab
Instances:   700
Attributes:  601
             [list of attributes omitted]
Test mode:   user supplied test set:  size unknown (reading incrementally)

=== Classifier model (full training set) ===

LibSVM wrapper, original code by Yasser EL-Manzalawy (= WLSVM)

Time taken to build model: 0.4 seconds

=== Evaluation on test set ===

Time taken to test model on supplied test set: 0.3 seconds

=== Summary ===

Correctly Classified Instances         301              100     %
Incorrectly Classified Instances         0                0     %
Kappa statistic                          1
Mean absolute error                      0
Root mean squared error                  0
Relative absolute error                  0      %
Root relative squared error              0      %
Total Number of Instances              301

=== Detailed Accuracy By Class ===

                 TP Rate  FP Rate  Precision  Recall   F-Measure  MCC      ROC Area  PRC Area  Class
                 1,000    0,000    1,000      1,000    1,000      1,000    1,000     1,000     1
                 1,000    0,000    1,000      1,000    1,000      1,000    1,000     1,000     2
                 1,000    0,000    1,000      1,000    1,000      1,000    1,000     1,000     3
Weighted Avg.    1,000    0,000    1,000      1,000    1,000      1,000    1,000     1,000

=== Confusion Matrix ===

   a   b   c   <-- classified as
  62   0   0 |   a = 1
   0  92   0 |   b = 2
   0   0 147 |   c = 3
```

Supplementary Figure S4 | Results for Machine Learning test in WEKA software[2], Version 3.8. The entry is SWeeP vectors with 600 coordinates; Class 1 represents organisms of genus *Corynebacterium*; class 2, *Klebsiella*; and class 3 *Escherichia*. All instances were classified correctly in the test set - 301 instances not used for training the model (see confusion matrix).

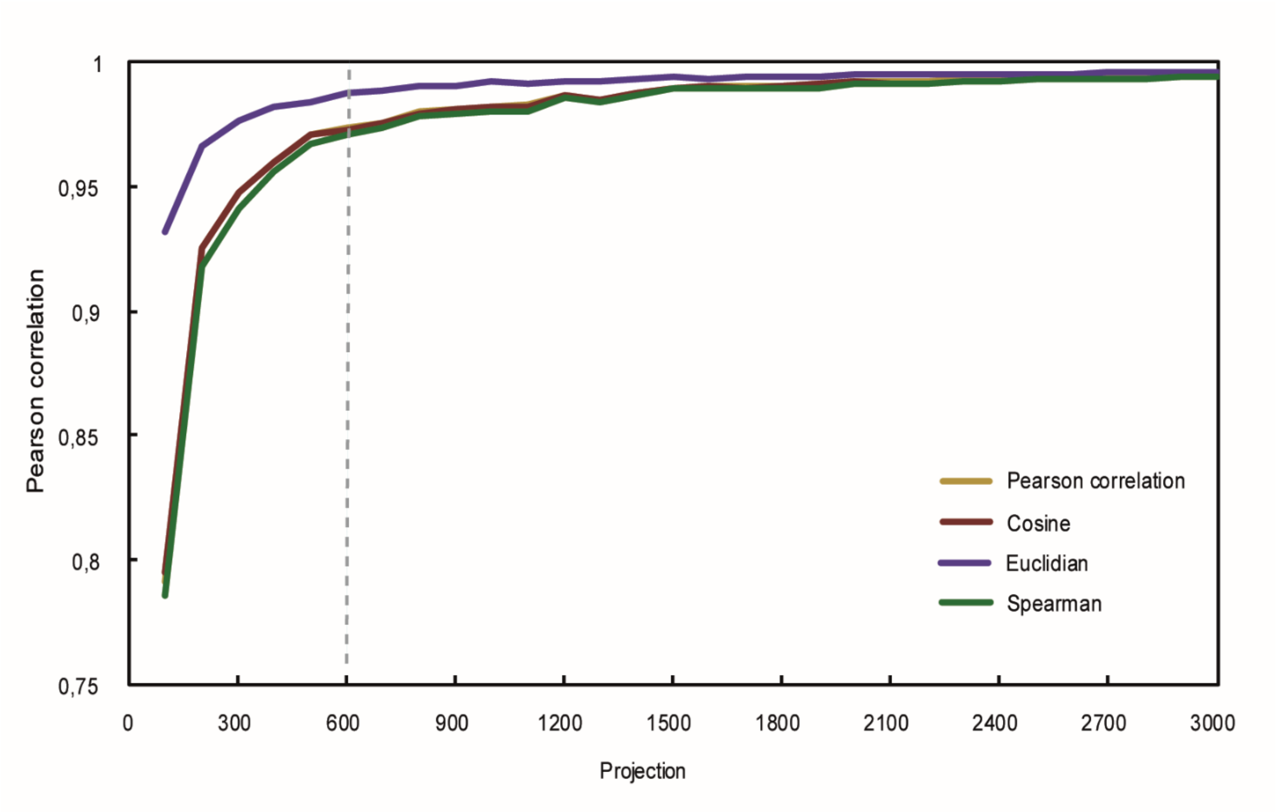

Supplementary Figure S5 | Pearson correlations between projections and HDV (Higher-dimension vector) for different projection distances and lengths for the mitochondrial proteomes. Different methods for calculating distances are indicated by different colors; Pearson correlation (yellow), cosines (dark red), Euclidian distance (purple), and Spearman correlation (green). The resulting vectors in *W*, without a dimensionality reduction and with the mask ("11011"), have sizes equal to 1.6×10^5. We analyzed 30 different-sized SWeeP projections between 100 and 3000 coordinates (at 100-coordinate intervals), checking the Pearson correlation between the distance matrix of the complete model and the distance matrix of the respective projections. Using 98% as the minimum correlation, we could have chosen any projection above the 400-coordinate size because the correlation at this point was 98.1% (see Supplementary Table S5).We chose the 600-coordinate projection because the analyzed branches were better distributed during the manual curation of the phylogenetic trees, and our goal was to identify the smallest projection that maintained quality for the mitochondrial dataset being analyzed. Nonetheless, for different datasets, different projections must be considered because the model enables this choice.

Supplementary Table S1| Construction time of SWeeP projections

Projection size in coordinates	Vector construction (sec)
No reduction (160K)	261.6
200	8
400	16
600	27
800	37.7
1000	48.25
1200	57.25
1400	72.15
1600	86.24
1800	101.80
2000	104.67
2200	117.29
2400	133.01
2600	146.46
2800	165.32
3000	177.74

Supplementary Table S2| Main methods of alignment free for phylogenetic analysis and/or sequence comparison purpose.

Tools	Purpose	Sequence type	Method	Authors and year of publication	Available (Y/N)
ACS	P	NT/AA	Measure of pairwise distances between sequences based on computing the average lengths of maximum common substrings, implemented by algorithm that uses suffix arrays.	Ulitsky et al (2006)[3]	Y
SlopeTree	P	AA	It is a method of phylogeny for whole genomes that estimates evolutions by measuring the decal of the correspondences of exact substrings as a function of the size of the match.	Bromberg, Grishin, Otwinowski (2016)[4]	Y
Andi	P	NT	Refers to a distance measure that is based on local alignments, which are anchored by means of unique maximum combinations of a minimum length, and correspondences can be searched using suffix arrays.	Haubold, Klötzl, Pfaffelhuber (2015)[5]	Y
IC–PIC	P	NT	Based on the quantification of information correlation (IC) and partial correlation of information (PIC) between nucleotides in a DNA sequence, to construct a vector that stores this information. The vector is used as identifier for a set of pairwise tests.	Gao, Luo (2012)[6]	Y
Multiple encoding vector	P	NT	Numerical vectors based on three chemical and physical properties to convert the nucleotide sequence into a new sequence, consisting of two types of letters. The number, mean position and letter position variation in each sequence is calculated.	Li et al (2017)[7]	N
TUP	P	NT	Based on FFP method. TUP Vector uses windows of length 3, which are not superimposed, to scan the DNA and represent the relative frequency distribution of the 64 trinucleotides.	Chen et al (2016)[8]	Y
UA	P	NT	The method is based on the classification of common and irrelevant subwords. The subwords of the smallest set are classified according to priority and the corresponding statistics are captured, removing the overlaps.	Comin, Verzotto (2012)[9]	Y
ALFRED-G	P	NT/AA	Based on ASC. It is a distance estimator for phylogeny reconstruction to calculate the lengths of common strings with pairing errors.	Thankachan et al (2017)[10]	Y

DLTree	P	NT/AA	Based on dynamic language model, DLTree is applied for phylogenetic analyzes based on complete genomes.	Wu, Yu, Yang (2017)[11]	Y
JD2Stat	P	NT/AA	Based on D2 statistics to extract k-mers and to generate pairwise distance, being able to be used for phylogenetic inference	Chan et al (2014)[12]	Y
LifePrint	P	NT/AA	Based on k-tuples of length 9, according to pre-defined criteria of substitution, blocks and refining, which are dependent on randomized process.	Reyes-Prieto et al (2011)[13]	Y
CVTree	P	NT/AA	An alignment-free and parameter-free phylogenetic tool using composition vectors (CVs) inferred from whole genome data.	Qi et al. (2004)[14]	Y
Multi-SpaM	P	NT	Word-based phylogeny approach based on multiple sequence comparison and Maximum Likelihood.	Dencker et al (2018)[15]	Y
FSWM	P	NT	Filtered Spaced Word Matches to estimate phylogenetic distances between large genomic sequences, based on gap-free local alignments with matching nucleotides at the match positions and with mismatches allowed at the don't-care positions.	Leimeister et al (2017)[16]	Y
Information – based network	S	AA	This approach analyzes the multivariate relationships of proteins using the probability distribution of amino acids to model the universe of proteins from a network.	Wan, Zhao, Yao (2017)[17]	N
SSAW	S	NT	It extracts k-mers from a sequence, then maps each k-mer to a complex number field. Series of complex numbers formed are transformed into feature vectors using the stationary discrete wavelet transform.	Lin et al (2018)[18]	Y*
k-mer spectrum	S	NT	Development of a measure of matched metagenomic dissimilarity based on the k-mer spectrum, useful for metagenomes.	Dubinkina et al (2016)[19]	Y
Kmacs	S	NT/AA	Based on ACS1. Longer common substrings are considered with k incompatibilities. It is a greedy heuristic to approximate the length of these substrings of incompatibility of k, based on generalized suffix matrices.	Leimeister, Morgenstern (2014)[20]	Y
Spaced-word	S	NT/AA	The method proposes the use of spaced k-mers, k-mers without pre-defined fixed positions, to compare the similarity between sequences, using a distance measure.	Boden et al (2013)[21]	Y
MissMax	S	NT/AA	Computes the most common substring with differences between each suffix of a sequence. This statistic is useful for calculating two measures of similarity: the	Pizzi (2016)[22]	Y*

			longest common substring and the average with k mismatches.		
Prot-SpaM	S	AA	Based on filtered spaced word matches (FSWM), using gap-free pairwise alignments of fixed-length words with matching amino-acid residues at certain pre-defined positions	Leimeister et al (2018)[23]	Y
FFP	S	NT/AA	Based on counting the frequencies of each characteristic of each genome by a sliding window. Counts are tabulated and normalized to generate a probability distribution vector.	Sims et al (2009)[24]	Y
K-mer natural vector	S	NT	Based on K-mer. The natural k-mer vector is the result of the concatenation of the parameters obtained by the occurrence frequency of each k-mer in the sequence and the average distance of each k-mer. The central moments are normalized.	Wen et al (2014)[25]	N
Natural vector	S	NT	The natural vector is the result of concatenation of the number assigned to each base and the mean value of the total distance of each base at the normalized center moments. A natural vector is used to obtain a numerical characterization of the DNA sequence.	Deng et al (2011)[26]	Y*
BioVec	S	NT/AA	Support vector machine to represent biological sequences with a single dense n-dimensional vector, based on n-gram.	Asgari et al (2015)[27]	Y
SWeeP	S	NT/AA	SWeeP uses the spaced-words concept to scan sequences and generate indices, which will be employed to create a high-dimensional vector allowing a reduction in dimensionality upon its projection onto a lower-dimensional vector, maintaining most of the comparison information.	-	Y

P - phylogenetic analysis, S - sequence comparison, NT – nucleotide, AA - amino acid protein, Y - Yes, N – No, *Software/source code available upon request.

Supplementary Table S3 | Inclusion criteria of alignment-free tools

Criteria	Details
a. Publicly available	Tools that are available upon request have been rejected due to lack of feedback.
b. Not only useful for phylogeny	Sweep is a general-purpose tool for sequence comparison and the analysis of phylogenetic trees was performed to explore the method, therefore, tools with scope only for phylogenetic analyzes were rejected from the analyzes.
c. Accepts input files in amino acid format	Due to the fact that we have chosen as the test set amino acid sequences, the tools that have input only to nucleotide sequences have been rejected.
d. Published in the last 5 years	Previously published tools have already been extensively tested by several authors[10-23-28-29], thus, only tools considered current are selected for the tests.

Supplementary Table S4 | Organisms and NCBI reference sequences used for the construction of heatmaps.

Organism	NCBI reference sequence	Publication
Corynebacterium pseudotuberculosis C231	NC_017301.1	Ruiz et al. (2011)[30]
Corynebacterium ulcerans str 809	NC_017317.1	Trost et al. (2011)[31]
*Klebsiella pneumoniae HS11286**	NC_016845.1	Liu et al. (2012)[32]
Klebsiella variicola AT-22	NC_013850.1	Pinto-Tomás (2009)[33]
Escherichia coli CFT073	NC_004431.1	Welch et al. (2002)[34]
Escherichia coli str K12 substr MG1655	NC_000913.3	Riley et al. (2006)[35]

* Chromosome sequence

Supplementary Table S5| Pearson correlations between the projections and the HDV at different projection distances and lengths for the mitochondrial proteomes.

**Projection size in coordinates**	**Distance Method**			
	**Pearson**	**Cosine**	**Euclidean**	**Spearman**
100	0.7909	0.7948	0.9317	0.7854
200	0.9257	0.9254	0.9665	0.9182
300	0.9472	0.9472	0.9761	0.9413
400	0.9597	0.9593	0.9818	0.9562
500	0.9704	0.9704	0.9842	0.9672
600	0.9733	0.9726	0.9872	0.9704
700	0.9759	0.9758	0.9885	0.9732
800	0.9801	0.9796	0.9901	0.9785
900	0.9815	0.9809	0.9904	0.9796
1000	0.9824	0.9823	0.992	0.9804
1100	0.9826	0.9817	0.991	0.9805
1200	0.9869	0.9864	0.9922	0.9855
1300	0.9851	0.9847	0.992	0.9837
1400	0.9879	0.9878	0.9935	0.9867
1500	0.9897	0.9891	0.994	0.9891
1600	0.9902	0.9902	0.9932	0.989
1700	0.99	0.9896	0.9938	0.9893
1800	0.9903	0.9902	0.994	0.9892
1900	0.9908	0.9911	0.9944	0.9896
2000	0.9921	0.9918	0.9948	0.9914
2100	0.992	0.9911	0.9951	0.9914
2200	0.9921	0.9916	0.9951	0.9912
2300	0.9931	0.9925	0.9952	0.9923
2400	0.9929	0.9926	0.9954	0.992
2500	0.9936	0.9932	0.9949	0.9928
2600	0.9935	0.9931	0.9955	0.9929
2700	0.9937	0.9934	0.9958	0.9929
2800	0.9937	0.9934	0.9957	0.9928
2900	0.9946	0.994	0.9959	0.994
3000	0.9947	0.9943	0.9959	0.9941

Note: Pearson's correlations in the pairwise distance analyses of the distance methods (Pearson's correlation, Euclidean distance, and Spearman's correlation) and the vectors arising from the projections of different sizes.

References

1. Hatrath, P. R. S. C. Jungers et al. 1997 (Phalangeal curvature Sloths) Evolution.pdf. **94**, 11998–12001 (1997).
2. Hall, M. *et al.* The WEKA Data Mining Software : An Update. **11**, 10–18 (2000).
3. Ulitsky, I., Burstein, D., Tuller, T. & Chor, B. The Average Common Substring Approach to Phylogenomic Reconstruction. *J. Comput. Biol.* **13**, 336–350 (2006).
4. Bromberg, R., Grishin, N. V. & Otwinowski, Z. Phylogeny Reconstruction with Alignment-Free Method That Corrects for Horizontal Gene Transfer. *PLoS Comput. Biol.* **12**, 1–39 (2016).
5. Haubold, B., Klötzl, F. & Pfaffelhuber, P. Andi: Fast and accurate estimation of evolutionary distances between closely related genomes. *Bioinformatics* **31**, 1169–1175 (2015).
6. Gao, Y. & Luo, L. Genome-based phylogeny of dsDNA viruses by a novel alignment-free method. *Gene* **492**, 309–314 (2012).
7. Li, Y., He, L., Lucy He, R. & Yau, S. S. T. A novel fast vector method for genetic sequence comparison. *Sci. Rep.* **7**, 1–11 (2017).
8. Chen, S. *et al.* Phylogenetic tree construction using trinucleotide usage profile (TUP). *BMC Bioinformatics* **17**, (2016).
9. Comin, M. & Verzotto, D. Alignment-free phylogeny of whole genomes using underlying subwords. *Algorithms Mol. Biol.* **7**, 1–12 (2012).
10. Thankachan, S. V., Chockalingam, S. P., Liu, Y., Krishnan, A. & Aluru, S. A greedy alignment-free distance estimator for phylogenetic inference. *BMC Bioinformatics* **18**, 1–8 (2017).
11. Wu, Q., Yu, Z. G. & Yang, J. DLTree: Efficient and accurate phylogeny reconstruction using the dynamical language method. *Bioinformatics* **33**, 2214–2215 (2017).
12. Chan, C. X., Bernard, G., Poirion, O., Hogan, J. M. & Ragan, M. A. Inferring phylogenies of evolving sequences without multiple sequence alignment. *Sci. Rep.* **4**, (2014).
13. Reyes-Prieto, F. *et al.* Lifeprint: A novel k-tuple distance method for construction of phylogenetic trees. *Adv. Appl. Bioinforma. Chem.* **4**, 13–27 (2011).
14. Qi, J., Luo, H. & Hao, B. CVTree: A phylogenetic tree reconstruction tool based on whole genomes. *Nucleic Acids Res.* **32**, 45–47 (2004).
15. Otu, H. H. & Sayood, K. A new sequence distance measure for phylogenetic tree construction. *Bioinformatics* **19**, 2122–2130 (2003).
16. Leimeister, C. A., Sohrabi-Jahromi, S. & Morgenstern, B. Fast and accurate phylogeny reconstruction using

filtered spaced-word matches. *Bioinformatics* **33**, 971–979 (2017).

17. Wan, X., Zhao, X. & Yau, S. S. T. An information-based network approach for protein classification. *PLoS One* **12**, 1–21 (2017).

18. Lin, J., Wei, J., Adjeroh, D., Jiang, B. H. & Jiang, Y. SSAW: A new sequence similarity analysis method based on the stationary discrete wavelet transform. *BMC Bioinformatics* **19**, 1–11 (2018).

19. Dubinkina, V. B., Ischenko, D. S., Ulyantsev, V. I., Tyakht, A. V. & Alexeev, D. G. Assessment of k-mer spectrum applicability for metagenomic dissimilarity analysis. *BMC Bioinformatics* **17**, 1–11 (2016).

20. Leimeister, C. A. & Morgenstern, B. Kmacs: The k-mismatch average common substring approach to alignment-free sequence comparison. *Bioinformatics* **30**, 2000–2008 (2014).

21. Boden, M. *et al.* Alignment-free sequence comparison with spaced k-mers. *OASIcs-OpenAccess Ser. Informatics* **34**, 24–34 (2013).

22. Pizzi, C. MissMax: Alignment-free sequence comparison with mismatches through filtering and heuristics. *Algorithms Mol. Biol.* **11**, 1–10 (2016).

23. Leimeister, C. A. *et al.* Prot-SpaM: fast alignment-free phylogeny reconstruction based on whole-proteome sequences. *Gigascience* **8**, 1–14 (2018).

24. Sims, G. E., Jun, S.-R., Wu, G. A. & Kim, S.-H. Alignment-free genome comparison with feature frequency profiles (FFP) and optimal resolutions. *Proc. Natl. Acad. Sci.* **106**, 2677–2682 (2009).

25. Wen, J., Chan, R. H. F., Yau, S. C., He, R. L. & Yau, S. S. T. K-mer natural vector and its application to the phylogenetic analysis of genetic sequences. *Gene* **546**, 25–34 (2014).

26. Deng, M., Yu, C., Liang, Q., He, R. L. & Yau, S. S. T. A novel method of characterizing genetic sequences: Genome space with biological distance and applications. *PLoS One* **6**, (2011).

27. Asgari, E. & Mofrad, M. R. K. Continuous distributed representation of biological sequences for deep proteomics and genomics. *PLoS One* **10**, 1–15 (2015).

28. Horwege, S. *et al.* Spaced words and kmacs: Fast alignment-free sequence comparison based on inexact word matches. *Nucleic Acids Res.* **42**, 7–11 (2014).

29. Jun, S.-R., Sims, G. E., Wu, G. A. & Kim, S.-H. Whole-proteome phylogeny of prokaryotes by feature frequency profiles: An alignment-free method with optimal feature resolution. *Proc. Natl. Acad. Sci.* **107**, 133–138 (2010).

30. Dobroviczká, T. *et al.* Effects of cadmium and arsenic ions on content of photosynthetic pigments in the leaves of Glycine max (L.) Merrill. *Pakistan J. Bot.* **45**, 105–110 (2013).

31. Trost, E. *et al.* Comparative analysis of two complete Corynebacterium ulcerans genomes and detection of

candidate virulence factors. *BMC Genomics* **12**, (2011).

32. Liu, P. *et al.* Complete genome sequence of Klebsiella pneumoniae subsp. pneumoniae HS11286, a multidrug-resistant strain isolated from human sputum. *J. Bacteriol.* **194**, 1841–1842 (2012).

33. Pinto-Tomás, A. A. *et al.* Symbiotic nitrogen fixation in the fungus gardens of leaf-cutter ants. *Science (80-.).* **326**, 1120–1123 (2009).

34. Welch, R. A. *et al.* Extensive mosaic structure revealed by the complete genome sequence of uropathogenic *Escherichia coli.PNAS;*1–5 (2002).

35. Riley, M. *et al.* Escherichia coli K-12: A cooperatively developed annotation snapshot - 2005. *Nucleic Acids Res.* **34**, 1–9 (2006).
